# Supplementary material for: Differential Activation Patterns in the Same Brain Region Led to Opposite Emotional States
Source: PLoS Biol. 2016 Sep 8;14(9):e1002546. doi: 10.1371/journal.pbio.1002546 (PMC5015828; doi:10.1371/journal.pbio.1002546)
Supplement: S1 Data — (DOCX) [file pbio.1002546.s001.docx]

When subjects from the higher- and lower-preference groups were asked what they tried to do during the induction period of the induction stage to increase the size of the feedback disk, the most common reply was: “I tried various things since I had no idea of the correct way.” Thus, we asked each subject to report the strategy that the subject thought was the most efficient to increase the size of the disk in detail. Details of the strategies that subjects reported were as follows. Note that these reports have been translated from Japanese into English:

Higher-preference group

Subject 1: “I tried to focus my attention on the fixation point at the center of the display.”

Subject 2: “I tried to imagine that I am doing various techniques for gymnastics.”

Subject 3: “I tried to remember contents of recent conversation with my friends in detail.”

Subject 4: “I tried to imagine that I am singing a song.”

Subject 5: “I tried to imagine that I am singing a song and dancing with a large number of people.”

Subject 6: “I tried to focus my attention on the fixation point at the center of the display.”

Subject 7: “I tried to imagine various colors.”

Subject 8: “I tried to translate recent daily happenings into English.”

Subject 9: “I tried to imagine that a face presented in the face period is moving.”

Subject 10: “I tried to imagine the details of a building in my junior high school.”

Subject 11: “I tried to remember items in a specific category (e.g., vegetable).”

Subject 12: “I tried to relax myself.”

Lower-preference group

Subject 13: “I tried to relax myself.”

Subject 14: “I tried to remember various characters in a famous video game.”

Subject 15: “I tried to remember my friend’s face which resembles a face presented in the face period.”

Subject 16: “I tried to count numbers, remember various natural scenes, and relax and keep on switching these strategies once in a while.”

Subject 17: “I tried to imagine that I am doing a back flip.”

Subject 18: “I tried to perform difficult numerical calculations.”

Subject 19: “I tried to relax myself.”

Subject 20: “I tried to imagine various scenes in my daily life.”

Subject 21: “I tried to imagine that I am listening to music.”

Subject 22: “I tried to imagine that I am listening to music.”

Subject 23: “I tried to remember detailed procedures in the experiment I conducted as an experimenter.”

Subject 24: “I tried to remember contents of classes I have recently attended in my university.”

No interview was conducted for the control group since they did not go through the induction stage with fMRI DecNef.
