# Supplementary figures and images for: Differential Activation Patterns in the Same Brain Region Led to Opposite Emotional States
Source: PLoS Biol. 2016 Sep 8;14(9):e1002546. doi: 10.1371/journal.pbio.1002546 (PMC5015828; doi:10.1371/journal.pbio.1002546)

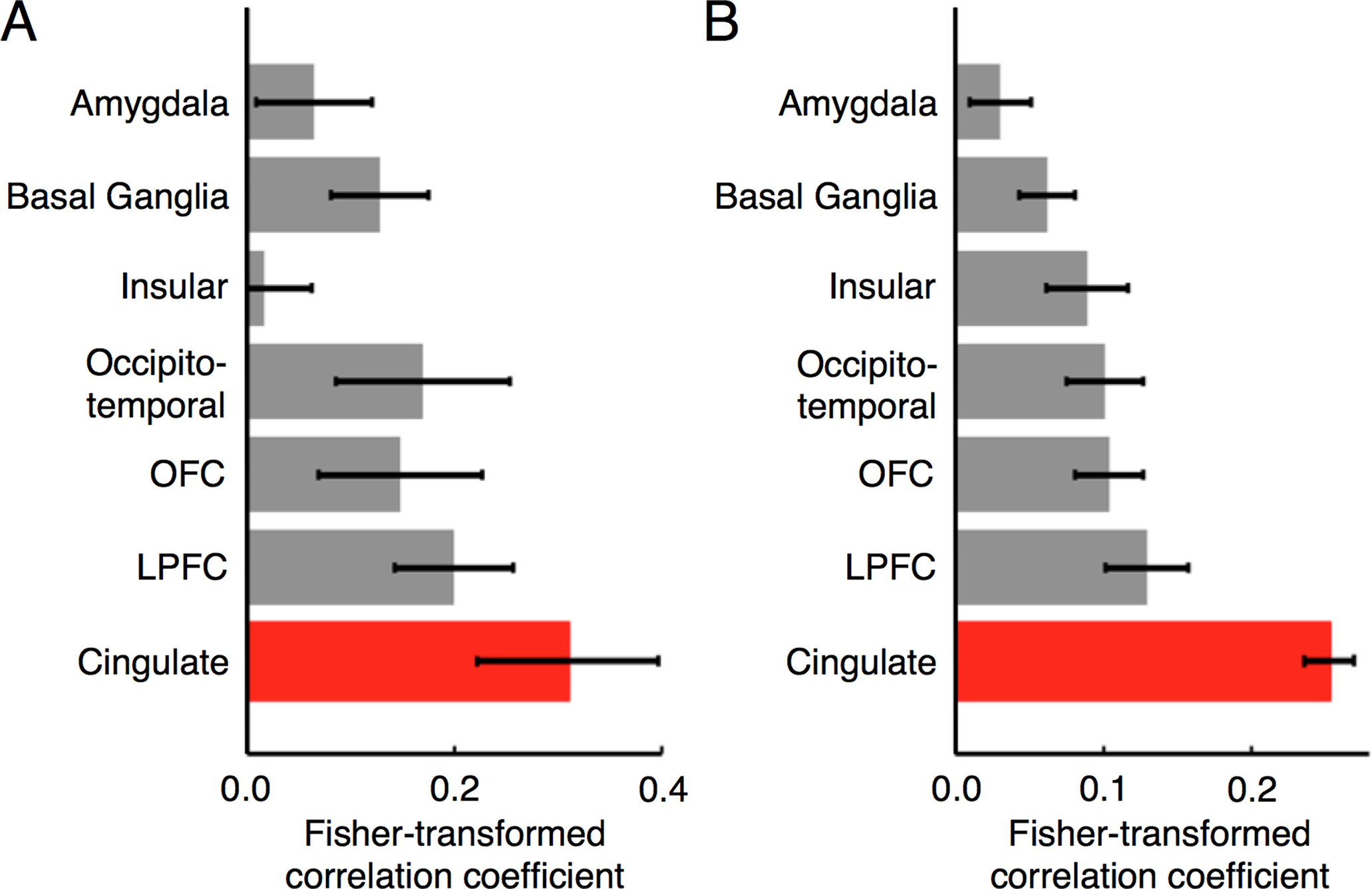

Supplement: S1 Fig — (A) The mean (± s.e.m.) Fisher-transformed correlation coefficient in the pilot experiment (n = 3). The CC showed the highest decoding performance. (B) The mean (± s.e.m.) Fisher-transformed correlation coefficient in the main experiment (n = 24). The pilot experiment (see Pilot Experiment in Materials and Methods for details) was aimed to select a single region that would be used as a target for fMRI DecNef in the main experiment. The selection was made based on comparison of performance of preference decoders among regions of interests (ROIs) that were implicated in facial preference [14–20]: the amygdala, basal ganglia, insular cortex, occipitotemporal cortex, orbit frontal cortex (OFC), lateral prefrontal cortex (LPFC), and cingulate cortex (CC) (see Pilot Experiment in Materials and Methods for the definition of the ROIs). Decoder’s performance for each ROI was defined as a correlation coefficient between subjects’ behavioral preference ratings and the estimated rating by the decoder from activation patterns of the ROI (see Pilot Experiment in Materials and Methods for details). In the main experiment (shown in B), we found that the decoder successfully predicted the subjects’ behavioral preference ratings based on the fMRI datasets measured in the fMRI decoder construction stage in the basal ganglia (one-sample two-tailed t-test on z-scores of the correlation coefficients after permutation, t23 = 3.20, p < 10−2; Bonferroni corrected; see Permutation Test in Materials and Methods for details of calculation of the z-scores), insular cortex (t23 = 3.07, p < 10−2, Bonferroni corrected), occipitotemporal cortex (t23 = 4.17, p < 10−3, Bonferroni corrected), OFC (t23 = 4.11, p < 10−3, Bonferroni corrected), LPFC (t23 = 4.46, p < 10−3, Bonferroni corrected), and CC (t23 = 12.52, p < 10−4, Bonferroni corrected), but not in the amygdala (t23 = 0.64, p = 0.53). The mean performance in the CC was significantly higher than that in the other ROIs (paired two-tailed t-test o [file pbio.1002546.s012.tif]

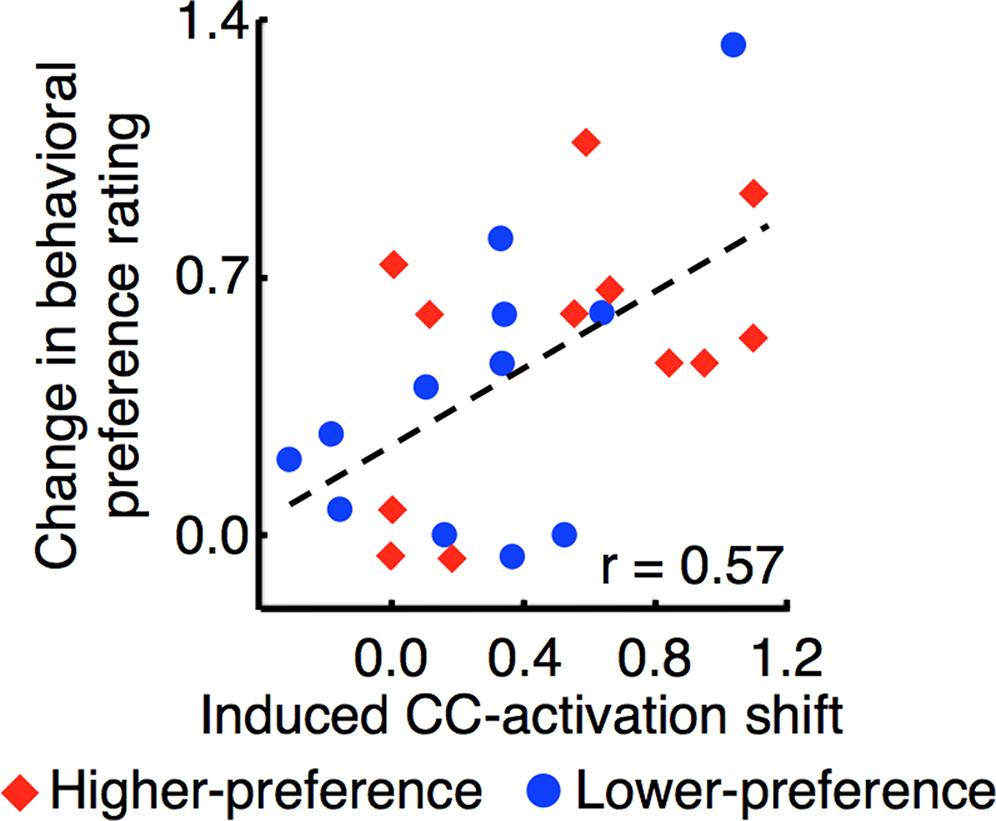

Supplement: S2 Fig — The same scatter plot of the induced CC-activation shift during the three-day induction stage versus the change in subjects’ behavioral preference rating for the higher- (red diamonds; n = 12) and lower- (blue circles; n = 12) preference groups as in Fig 4, except that the sign of the data for the lower-preference group was reversed. The broken line indicates the ordinary least-square regression. For each of the higher- and lower-preference groups, we found a significant correlation between the CC-activation shifts and changes in subjects’ behavioral preference rating (r = 0.62 for the higher-preference group, r = 0.50 for the lower-preference group, permutation test, p < 0.05; see Permutation Test in Materials and Methods for details of the permutation test). (TIF) [file pbio.1002546.s013.tif]

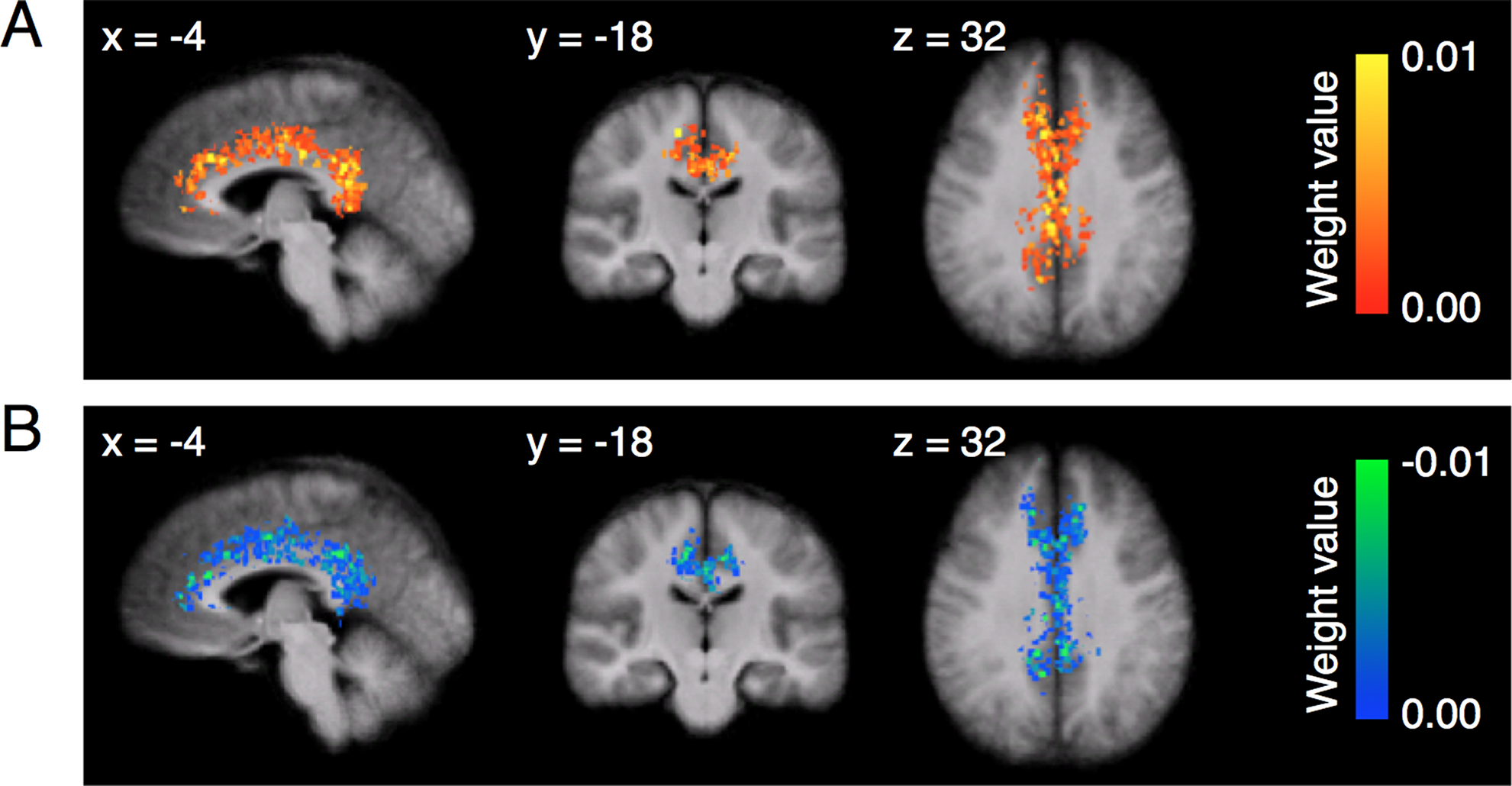

Supplement: S3 Fig — (A) The mean spatial distribution of the positive weight values across subjects. (B) The mean spatial distribution of the negative weight values across subjects. We tested whether the activation patterns for positive and negative facial preferences are spatially dissociable in the CC. We also tested whether the spatial activation pattern is consistent across subjects. In particular, we compared spatial structures of activation patterns associated with positive and negative facial preferences in two ways. The first analysis was aimed to examine spatial distributions of weights of the preference decoder in the CC. The preference decoder was constructed based on measured activation patterns in the CC in the fMRI decoder construction stage, during which subjects reported their preference rating to faces (see Materials and Methods for details). The preference decoder was constructed to estimate the subjects’ preference ratings based on the activation patterns in the CC. Thus, the CC voxels that have positive weight values contribute to positive facial preferences, whereas those that have negative weight values contribute to negative facial preferences. Therefore, examination of the spatial distributions of the weight values in the CC should tell us whether the activation patterns associated with positive and negative facial preferences are distributed differently in the CC. Positive (shown in A) and negative (shown in B) weight values uniformly distributed and their distributions were largely overlapped in the CC. Second, we tested whether positive and negative weight patterns are consistent across subjects. If these patterns are consistent across subjects, the mean weight value across subjects for a given voxel should be significantly different from zero. In contrast, if these patterns are not consistent across subjects, the mean weight value for a given voxel would be around zero. We found that the mean weight value across subjects was not significantly different from [file pbio.1002546.s014.tif]

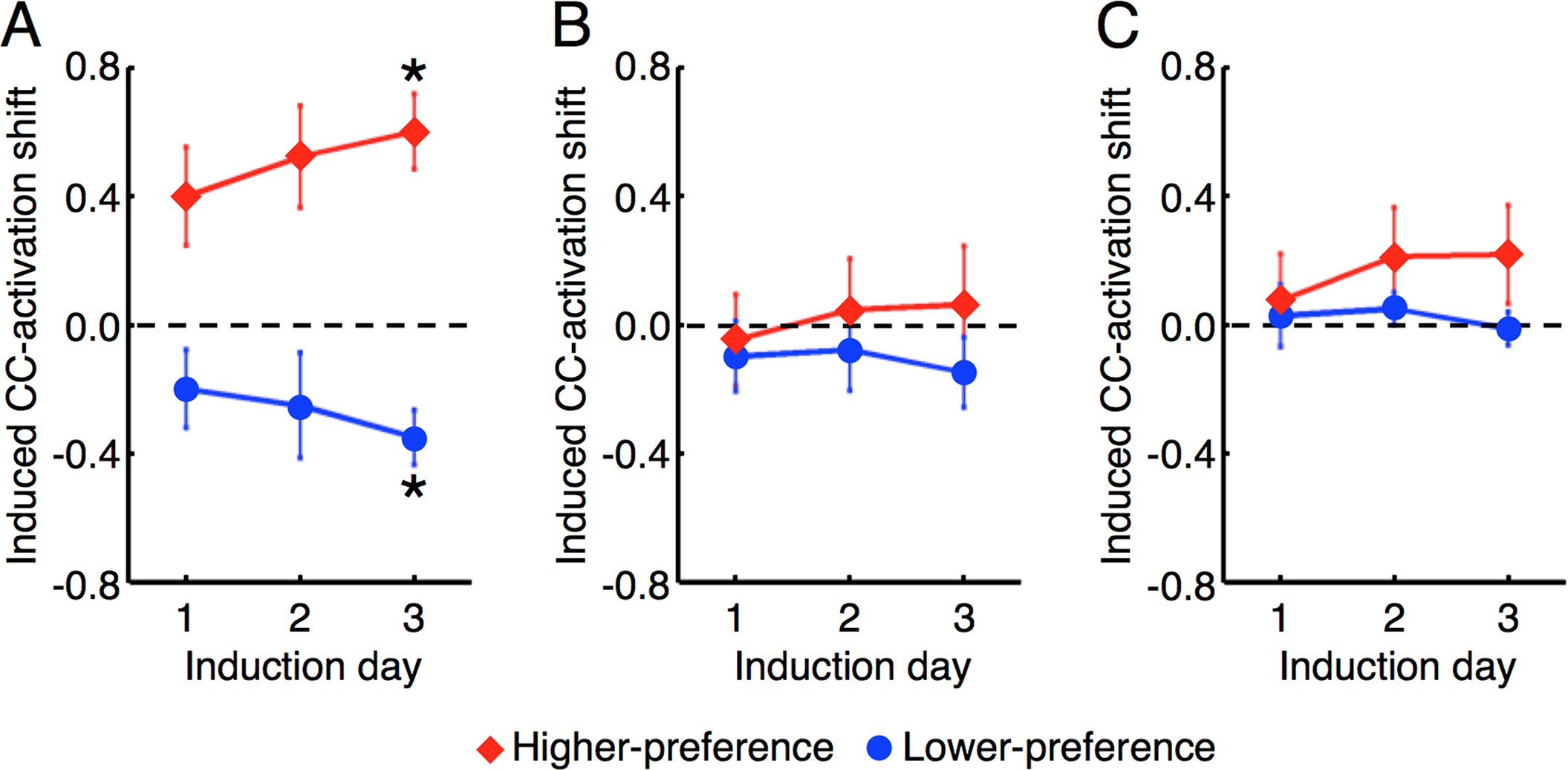

Supplement: S4 Fig — (A) Induction period. (B) Fixation period. (C) Inter-trial period. The mean (± s.e.m.) induced CC-activation shifts for the higher- (red) and lower- (blue) preference groups. To test whether similar activation patterns were induced in the CC during the fixation and inter-trial period to during the induction period, we calculated the induced CC-activation shifts over the three-day induction stage using the preference decoder and compared them among the induction, fixation, and inter-trial periods. If activation patterns were similar among these periods, the same pattern of induced CC-activation shifts should be observed for all three periods. A three-way mixed-model ANOVA with factors being period (induction, fixation, versus inter-trial periods), induction day (Day 1, Day 2, versus Day 3), and group (higher- versus lower-preference groups) was applied to the induced CC-activation shifts. The main effects of period (F2,44 = 3.48, p = 0.04) and group (F1,22 = 5.51, p = 0.03) were significant. Significant interactions were obtained between period and group (F2,44 = 15.09, p < 10−4) and between induction day and group (F2,44 = 3.48, p = 0.04). These results are not in accord with the possibility that the same pattern of induced CC-activation shifts should be observed for all three periods. As shown in (A), for the induction period, on Day 3, the induced CC-activation shifts were significantly greater than zero for the higher-preference group (one-sample two-tailed t-test, t11 = 5.15, p < 10−3; Bonferroni corrected) and significantly smaller than zero for the lower-preference group (t11 = 4.09, p < 10−2; Bonferroni corrected). These results demonstrate that by Day 3 subjects successfully learned to induce activation patterns in the CC that correspond to higher (higher-preference group) or lower (lower-preference group) preference ratings during the induction stage. During the fixation period (shown in B), for neither group the induced CC-activation shifts were significan [file pbio.1002546.s015.tif]

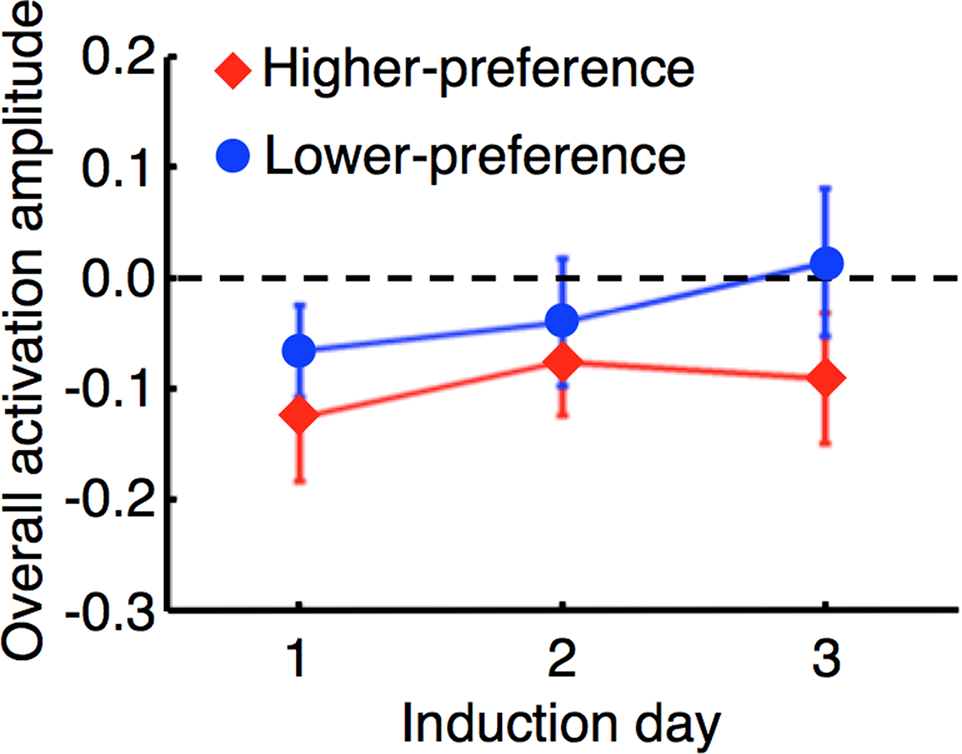

Supplement: S5 Fig — To test whether the overall mean amplitude in the CC changed during the induction period, we conducted an amplitude-based analysis using the general linear model on the BrainVoyager QX software. The amplitude of each voxel in the CC was calculated based on the contrast between the induction and inter-trial periods, and the calculated amplitudes were averaged across the voxels. To test if the overall amplitudes differ between the higher- and lower-preference groups, a two-way mixed-model ANOVA with factors being group (higher- versus lower-preference groups) and induction day (day 1, day 2, versus day 3) was applied to the overall amplitudes in the CC. None of a main effect of group (F1,22 = 1.01, p = 0.33), a main effect of induction day (F2,44 = 1.23, p = 0.30), or an interaction between the two factors (F2,44 = 0.45, p = 0.64) was significant. In addition, for neither group, the amplitudes were significantly different from zero on any day (one-sample two-tailed t-test, t11 < 2.17, p > 0.05). (TIF) [file pbio.1002546.s016.tif]
